# A Case of Functional, Simulating Organic Disease of the Heart

**Published:** 1843-08

**Authors:** H. R. Roberts

**Affiliations:** Columbia, Tennessee


### Art. VI.—*A case of Functional, Simulating Organic Disease of the Heart.* By H. R. Roberts, M.D., Columbia, Tennessee.

February 2d, 1843, I was requested to see Mr. A. D. Bailey, a citizen of Columbia, by trade a hatter, who had been taken ill about a week previously. I found him in the following condition as recorded at the time: Entirely free from pain, skin and expression of countenance natural, tongue clean, and appetite good, some little cough with free expectoration; accompanying these favorable symptoms were others of a different character. The action of the heart was exceedingly irregular, and occasionally rolling or bounding against the walls of the chest with alarming violence, then rapid, feeble and intermitting. The pulse was still more remarkable, and did not correspond entirely with the action of the heart: for a moment it would be too rapid to count, after which there would be an intermission succeeded by a few strokes that were slow and bounding; between each of these pulsations the sensation communicated to the finger was of a quivering or fluttering character, as if the blood had been thrown into a soft elastic tube; there was not sufficient tensity to give it a thrilling sensation. The artery was loose, and could be easily pushed out of its position and contorted with the two fingers into the shape of the italic *S*. The breathing was easy in any position, though there was a perceptible shortness of breath when talking, which would cause a by-stander to feel uneasy at the the termination of each sentence. I was called in to give an opinion relative to the pathology of the disease, and enquired particularly into the history of the case. I learned that about ten days previous to the time that I saw him, he had first discovered an irregularity in the heart's action, accompanied with some difficulty of breathing on lieing down at night, which was soon followed by an irritation in the throat, cough, febrile symptoms, &c. I also learned that on the preceding day, when he was first taken, he had eaten an enormously large quantity of a mixture composed of snow, cream, and sugar, and that he had been a good deal exposed to the severe and disagreeable weather that occurred about that time. And then there were other exciting causes which I was already aware of, and which I believed had no inconsiderable influence in bringing on this state of things. I knew him to be a man of strong intellect violent prejudices, great nervous mobility, and exceedingly sensitive; his mind had been about this period actively engaged in the discussion of an exciting question at a Mechanic's Society, and he had been forced by his creditors into bankruptcy, which his pride and extreme sensitivenes could not well bear.

As it was a matter of the utmost importance to determine whether the affection of the heart was merely functional, or whether the organ itself or its capsule was the seat of disease, I enquired of him if at any time preceding the present attack, he had been aware of an irregular action of the heart; if, on taking violent exercise, such as running, walking rapidly up hill, or ascending a flight of stairs, it had not been increased, or if there was not oppression and difficulty of breathing. He had not been aware of any, though he had often taken such exercise, until the day before he was confined to his bed; his pulse he thought had been uniformly natural, and I remembered myself to have examined it at one time during the last summer, when he thought he was threatened with an attack of fever, and there was nothing remarkable about it. As he could at this time easily get in and out of bed, I requested him to do so and suddenly to assume the erect and recumbent posture alternately. I found but slight if any alteration, either in the action of the heart or pulse; there had not been at any time a symptom of cerebral determination or congestion, no headache, tinnitus aurum, or stupor; though the pulsation of the carotids could be distinctly seen, there was none of that universal throbbing, with weight and tension, increased by stooping or resuming the recumbent position, so characteristic of hypertrophy; again there was not the full, firm, strong, and sharp pulse which belongs to that disease. As the attack was sudden, an acute inflammatory affection of the heart or the pericardium might be suspected. I pressed my fingers forcibly upon the intercostal spaces, requested him to bring a long breath, lie alternately upon the right and left side; no pain, uneasiness, or oppression was produced. There had been no disposition to syncope, no marked anxiety in the expression of the countenance; the fever had been mild; the cough, though at first dry, was now accompanied with free and easy expectoration; no pungent or lancinating pain in the precordial region, or shoulder and arm: neither carditis or pericarditis could exist under such circumstances. Had I been governed alone by the action of the heart and arteries, the disease might have been pronounced pericarditis in a sub-acute form, for the pulse was of that fluttering, irregular, and intermittent character which marks that disease, but there was no other symptom to corroborate it. On forcing the epigastrium upwards underneath the left hypochondrium, there was no pain or uneasines produced, not the slightest disposition to faintness, no dyspnœa or anxiety, and, as I have before observed, any position could be assumed in bed without difficulty. I do not profess to be an adept in that most valuable agent in diagnosis, auscultation, and I have to regret that I am not so; but the following were the physical signs present at the time that I saw him. By percussion on either side of the chest, if with any degree of force, there was a tickling or uneasy sensation produced, that I do not remember to have observed in any other case; it was not painful, but so unpleasant that he would not bear it often. There was but little difference in the sound on either side as high up as the clavicle; about the fourth or fifth rib on the left side it was slightly flat and dull. The range of the sound of the heart was extensive, and could be perceived over a large portion of the exterior aspect of the chest; the action was tumultuous, producing a general commotion rather than a forcible and circumscribed impulse. The respiratory sound was clearly perceptible on the whole of the left side of the chest, and on the right, except a circumscribed space of about six inches, and even there it may have been masked by the sound of the heart—over the sternum and along the course of the bronchia the moist crepitous ronchi were very distinct. I will just here remark that pressure on the epigastric region caused very considerable uneasiness; his appetite was voracious, indeed perfectly ungovernable.

Having made this examination, I was prepared to give an opinion, that the disease of the heart was functional, proceeding from nervous irritability, and sympathetic with a subacute inflammation, evidently existing in the mucous coat of the stomach and bronchia; and I regarded the immense quantity of the freezing mixture he had eaten, the depressing moral causes that were constantly operating upon a mind remarkably sensitive, quite sufficient to produce all the phenomena now observed. I told him that I did not regard the case as immediately dangerous, but that an action so violent and irregular could not long exist without producing an organic affection—most probably hypertrophy. At the time he did not wish to be put upon any course of treatment, and I left without recommending any. He continued a few days without improving and I was again called and requested to treat the case. Basing my treatment upon the pathology I have given, I enjoined perfect rest, forbid all exciting causes both physical and mental, and attempted (though I did not well succeed) the strictest course of abstemiousness. A blister had before been applied over the epigastric region, extending as high up as the fourth or fifth rib; I had it re-applied; and to quiet nervous irritability and check the cough which was somewhat distressing, gave a mixture composed of the extract of hyoscyamus and squills. I found him the next day a little more quiet, and he had rested pretty well during the night, expectorating freely; the same course was continued, with the addition of a small morphine pill at night, for several days; but the action of the heart was still rapid and irregular; cough improved veiy much. I now determined to try fully the efficacy of digitalis, and gave it in doses of thirty drops of the tincture, three times a day; the morphine still given at night. Under this treatment all the symptoms improved: the action of the heart became more regular and less rapid; cough had almost entirely disappeared; his appetite was now the most formidable thing I had to contend with, and it was astonishing what influence the slightest imprudence in eating exercised over the action of the heart. He was himself aware of the fact, but acknowledged his inability to govern it; a mild purgative would, however, generally afford relief. This course of treatment was pursued with slight variations for two or three weeks; in the meantime cups had been applied over the epigastrium, and a blister between the shoulders. He was now so much improved that he insisted on going to the country; as he would then be away from the scenes of his misfortunes, and the many depressing causes that were still operating, I consented for him to do so. He bore the ride of ten miles, and sent me word at night that he felt quite well; unfortunately for him there came on at this time the long cold spell of weather we had about the first of March; the house he was staying in was comparatively very open; his diet was not such as he had been accustomed to, and he very soon became dissatisfied. The symptoms began to grow worse; he took violent cold and had most distressing attacks of palpitation; for two weeks he had to bear this state of things; at the end of that time, however, he came to town very much exhausted, and evidently much worse. Under the influence of anodynes, however, he slept well the first night, but the next day I witnessed the most distressing scene. I found him propped up in bed, face livid, anxious expression of countenance, distressing dyspnœa, and arterial blood occasionally gushing from his mouth, the action of his heart tumultuous, and capillary circulation suspended. I now suspected that the right side of the heart had been enlarged. I saw that congestion of the lungs must be removed or death would in a few minutes be the inevitable result. The lancet was in my hand, but I could not bleed with a soft, feeble, fluttering pulse that would vanish upon the slightest pressure. All that was left for me to do then was to quiet, by whatever other means I had in my power, the spasmodic action of the heart, restore capillary circulation and induce the blood to the surface. No time was lost in administering such remedies as were calculated to quiet nervous irritability, and reduce spasmodic action; besides these, mustard plasters were applied to the wrist and ancles, friction on the legs and arms with dry mustard was kept up until sufficient excitement was produced to create a glow upon the surface; a belladonna plaster was also applied over the heart. In a short time the flurry began to subside; I now learned that he had eaten very imprudently that morning, and I attributed many of the distressing symptoms he had just had to that cause. To keep up the regular action a saline cathartic was administered; at night a hot pediluvium, followed by friction with dry mustard, accompanied with an anodyne draft, rendered him perfectly quiet, and he slept comparatively well.

The next day the same scene was partly enacted over again, though with much less distress. There was evidently great irritation in the bronchial tubes and their ramifications; a large blister was applied on the breast, and a saline draught given which operated well; hyoscyamus, squills, and laudanum, relieved greatly the irritation, and the cough together with the hemoptysis was gradually subsiding; after a day or two the digitalis was again used, a pill composed of calomel, squills, and ipecac., given night and morning—he now improved rapidly, and in a short time the action of the heart and pulse became almost natural. Moderate exercise was allowed, and so long as he could be prevailed upon to live lightly no unfavorable symptom was present; this however was almost impossible, and he was frequently under the necessity of taking remedies to relieve oppression in consequence of imprudence in eating. As he grew better his pulse increased in force and tensity, so much so as to require blood-letting two or three times. I left him attending to his ordinary affairs; and as his business at this time is of an exceedingly unpleasant character, well calculated to irritate and depress, a return of the malady may be reasonably calculated upon.

As this case was under my care nearly three months, during which time the treatment was often varied to suit each particular symptom, I could not in a report already made too long give it in detail. My object has been to show that, notwithstanding the disease in many of its features resembled an organic affection of the heart, it was strictly functional; and I have no hesitation in saying that the treatment which would have been indicated had it been organic, would in this case most certainly have proved fatal.

